# *Staphylococcus aureus* Isolates from Bovine Mastitis in Eight Countries: Genotypes, Detection of Genes Encoding Different Toxins and Other Virulence Genes

**DOI:** 10.3390/toxins10060247

**Published:** 2018-06-17

**Authors:** Valentina Monistero, Hans Ulrich Graber, Claudia Pollera, Paola Cremonesi, Bianca Castiglioni, Enriqueta Bottini, Alejandro Ceballos-Marquez, Laura Lasso-Rojas, Volker Kroemker, Nicole Wente, Inge-Marie Petzer, Carlos Santisteban, Jeff Runyan, Marcos Veiga dos Santos, Bruna Gomes Alves, Renata Piccinini, Valerio Bronzo, Mohamed Salah Abbassi, Meriam Ben Said, Paolo Moroni

**Affiliations:** 1 Department of Veterinary Medicine, University of Milan, via Celoria 10, 20133 Milan, Italy; valentina.monistero@gmail.com (V.M.); claudia.pollera@unimi.it (C.P.); renata.piccinini@unimi.it (R.P.); valerio.bronzo@unimi.it (V.B.); paolo.moroni@unimi.it (P.M.); 2 Agroscope, Research Division, Food Microbial Systems, Schwarzenburgstrasse 161, 3003 Bern, Switzerland; hansulrich.graber@agroscope.admin.ch; 3 Institute of Agricultural Biology and Biotechnology, National Research Council, via Einstein, 26900 Lodi, Italy; casti@ibba.cnr.it; 4 Laboratorio de Microbiologia Clinica y Experimental, Departamento de Sanidad Animal y Medicina Preventiva SAMP/CIVENTAN, Becaria CONICET, Facultad de Ciencias Veterinarias, Universidad Nacional del Centro de la Provincia de Buenos Aires (FCV, UNCPBA), Paraje Arroyo Seco S/N, Campus Universitario, CP 7000 Tandil, Buenos Aires, Argentina; bottini.enriqueta@gmail.com; 5 Laboratorio de Calidad de Leche y Epidemiología Veterinaria (Grupo CLEV), Universidad de Caldas, Calle 65 #26-10, Manizales, Caldas, Colombia; alejandro.ceballos@ucaldas.edu.co (A.C.-M.); laura.lasso.mvz@gmail.com (L.L.-R.); 6 Bioprocess Engineering—Faculty II, University of Applied Sciences and Arts, Microbiology Heisterbergallee 12, 30453 Hannover, Germany; Volker.Kroemker@hs-hannover.de (V.K.); nicole.wente@hs-hannover.de (N.W.); 7 Faculty of Veterinary Science, University of Pretoria, M35, Pretoria 0110, South Africa; Inge-Marie.Petzer@up.ac.za; 8 Quality Milk Production Services, Animal Health Diagnostic Center, Cornell University, 240 Farrier Road, Ithaca, NY 14850, USA; cgs1@cornell.edu (C.S.); jpr253@cornell.edu (J.R.); 9 Department of Animal Nutrition and Production, School of Veterinary Medicine and Animal Sciences, Rua Duque de Caxias Norte, 225, Pirassununga-SP 13635900, Brazil; mveiga@usp.br (M.V.d.S.); bgalves@usp.br (B.G.A.); 10 Tunisian Institute of Veterinary Research, University of Tunis El Manar, Tunis 1068, Tunisia; salahtoumi_mohamed@yahoo.com (M.S.A.); mbs-mariem@hotmail.fr (M.B.S.)

**Keywords:** mastitis, dairy cow, *S. aureus*, genotypes, virulence genes

## Abstract

*Staphylococcus aureus* is recognized worldwide as one of the major agents of dairy cow intra-mammary infections. This microorganism can express a wide spectrum of pathogenic factors used to attach, colonize, invade and infect the host. The present study evaluated 120 isolates from eight different countries that were genotyped by RS-PCR and investigated for 26 different virulence factors to increase the knowledge on the circulating genetic lineages among the cow population with mastitis. New genotypes were observed for South African strains while for all the other countries new variants of existing genotypes were detected. For each country, a specific genotypic pattern was found. Among the virulence factors, *fmtB*, *cna*, *clfA* and leucocidins genes were the most frequent. The *sea* and *sei* genes were present in seven out of eight countries; *seh* showed high frequency in South American countries (Brazil, Colombia, Argentina), while *sel* was harboured especially in one Mediterranean country (Tunisia). The *etb*, *seb* and *see* genes were not detected in any of the isolates, while only two isolates were MRSA (Germany and Italy) confirming the low diffusion of methicillin resistance microorganism among bovine mastitis isolates. This work demonstrated the wide variety of *S. aureus* genotypes found in dairy cattle worldwide. This condition suggests that considering the region of interest might help to formulate strategies for reducing the infection spreading.

## 1. Introduction

*Staphylococcus aureus* continues to be one of the most prevalent pathogens causing intramammary infections (IMI) in dairy cows. It’s a worldwide pathogen recognized as a cause of subclinical infections, resulting in increased somatic cell count (SCC), but may also cause clinical mastitis. Staphylococcal mastitis is a major problem in dairy industry, affecting animal health and causing economic losses of up to €300 per cow per year, due to the reduced milk quality and production [1,2]. The main reservoir of *S. aureus* seems to be the infected quarter, and transmission usually occurs from cow to cow during milking.

Successful infection depends on virulence factors produced by *S. aureus.* A wide spectrum of secreted and cell surface-associated virulence factors can be expressed to promote adhesion to the host extracellular matrix components, damage host cells, and fight the immune system [3]. At least 25 different toxins (such as enterotoxins SEA to SEQ, toxic shock syndrome toxin-1 TSST-1, exfoliative toxins Eta, Etb), 15 microbial surface components recognizing adhesive matrix molecules, which are important for adhesion to tissues (such as clumping factor A *clfA*, intercellular adhesion genes *icaA* and *icaD*), 20 immune evasion molecules (such as protein A, coagulase, haemolysins and leucocidins, factors associated with suppressing innate immunity) and several other *S. aureus* virulence factors are known. Some virulence factors are expressed by genes that are located on mobile genetic elements called pathogenicity islands (i.e., TSST and some enterotoxins) or lysogenic bacteriophages (i.e., Panton-Valentine Leucocidin, PVL) and others such as the staphylococcal complement inhibitor, *scn*, the chemotaxis inhibitory protein, *chp*, and staphylokinase, *sak*, are integrated in the bacterial chromosome [4]. Furthermore, *S. aureus* can also acquire the staphylococcal cassette chromosome SCC*mec*, giving rise to methicillin-resistant *S. aureus* (MRSA) [5]. In fact, the expression of the *mecA* or *mecC* gene in *S. aureus* confers resistance to most of β-lactams, drugs which are frequently used for treatment of mastitis [6].

The determination of the origin of the *S. aureus* isolates involved in the aetiology of bovine mastitis is highly relevant from the epidemiological point of view. In such a context, the precise characterization of this pathogen provides monitoring of the bacterial strains dissemination among animal populations.

Over the past two decades, a wide range of phenotyping and genotyping methods have been used or developed for *S. aureus* including, but not limited to, ribotyping, RAPD-typing, PFGE, MLST, spa-typing, RS-PCR, coagulase gene RFLP, MLVA, micro-arrays and whole genome comparisons [7,8,9,10,11]. Many molecular epidemiological studies have been based on the use of selected targets in the genome, giving rise to banding patterns based on restriction- or primer binding sites, or to allelic profiles for housekeeping or virulence genes [12]. Such studies continue to be useful diagnostic tools when the aim is to understand pathogen sources and transmission mechanisms. Moreover, among the genotyping methods, the RS-PCR, based on amplifying the 16S-23S rRNA intergenic spacer region by PCR (ribosomal spacer-PCR), showed to be accurate, rapid and inexpensive with a discriminatory power like the other more-recognized genotyping methods [13].

The aim of this study was to genotype by RS-PCR and compare the molecular-epidemiologic profiles of a large world collection of *S. aureus* isolates to deepen the knowledge on the circulating genetic lineages among the cow population with mastitis. The isolates were investigated for three genes related to host adhesion and invasion (*clfA*, clumping factor; *cna*, collagen-binding protein; *fmtB*, cell wall-associated protein), 22 genes that have the potential to interfere with host defence mechanisms (*tsst*, toxic shock syndrome toxin-1; *scn*, staphylococcal complement inhibitor; *chp*, chemotaxis inhibitory protein; *sak*, staphylokinase; enterotoxins from *sea* to *sel*; exfoliative toxins *eta*, *etb* and leucocidins *lukE*, *lukE-lukD*, *lukM*, *lukSF-PV*), and the gene encoding the acquisition of methicillin resistance (*mecA*).

## 2. Results

In this study, a total of 120 isolates collected from eight different countries were genotyped by RS-PCR and analyzed for 26 virulence factors related to *S. aureus* pathogenicity, such as genes related to host adhesion and invasion (*clfA*, *cna*, *fmtB*), genes that have the potential to interfere with host defense mechanisms (*tsst*, *scn*, *chp*, *sak*, enterotoxins from *sea* to *sel* and leukotoxins), and the gene encoding the acquisition of methicillin resistance (*mecA*).

### 2.1. RS-PCR Genotyping

For the RS-PCR genotyping analysis, the genotypes, were named and extended according to a previous study [14] leading to the genotypes GTA to GTZ, followed by the genotypes GTAA to GTAZ, GTBA to GTBZ, and so on. A genotypic variant, differing in only 1 band of a known genotype, was indicated with roman numerals superscripted after the name of the genotype (e.g., GTR^I^, GTR^II^). Variation in more than one band, between profiles, was regarded as a new genotype. Finally, genotypes and their variants (e.g., genotype GTB and its variants GTB^I^, GTB^II^, GTB^III^), encompassing at least 5% of all the strains, were combined into genotypic clusters (CL).

**Figure 1:**
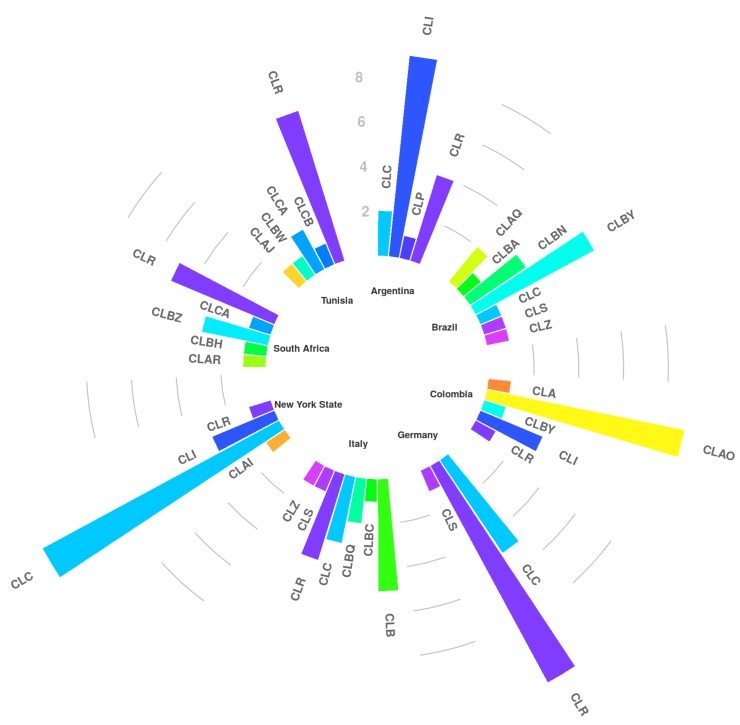
Representation of the major genotypes with their variants combined into genotypic clusters (CL).

New genotypes comprising GTAR, GTBZ, and GTCA were observed for South African and Tunisian strains (Table 1). For all the other countries, at maximum new variants of existing genotypes were detected. They included GTI^V^, GTI^VI^ (Argentina), GTAQ^I^, GTBN^I^, GTBN^II^, GTBY^I^ (Brazil), GTAO^I^, GTAO^II^ (Colombia), GTR^XIII^ (Italy), GTC^V^ and GTI^V^ (New York State). For each country, a specific genotypic pattern was found. Major genotypes with their variants were combined into genotypic clusters (CL) [14] and showed in Figure 1. For Argentina (Table 1, Figure 1) it mainly consisted of CLI (56% of GTI variants) and CLR (25% of GTR variants), whereas for Brazil CLBN (20% of GTBN plus variants) and CLBY (40% of GTBY plus a variants) were most prominent. The Colombian strains were mainly positive for GTAO and its variants (CLAO, 60%). In the case of Germany and Italy, the most prevalent genotypes were GTC^I^, GTR plus variants, and GTB, combined into CLC (30%), CLR (64.7%) and CLB (29.4%), respectively. Finally, the main genotypes observed for the South African and Tunisian strains were GTR and its variants (CLR, 45%), whereas the American strains were mainly positive for GTC and variants of it (CLC, 70.6%). In conclusion, cluster C was observed mostly in Germany and New York State, while CLR was widely disseminated in seven countries; especially it was frequently detected in Argentina, Germany, Italy, South Africa and Tunisia but less in Colombia and New York State.

All the existing genotypes including their variants such as GTC and GTC^I^ had been previously isolated from bovine intramammary infection or bovine milk. Exceptions were GTBH (sandwich with Mozzarella) and GTAO (human nasal carriage).

### 2.2. Virulence Genes

All the 120 isolates analyzed in this study were positive for coagulase (*coa*) and thermonuclease (*nuc*) genes, but negative for a gene involved in host cell invasion, the exfoliative toxin (*etb*), and for SEB and SEE enterotoxins. The distribution of the virulence genes for each country is described in detail below.

Dendograms derived from the combination between RS-PCR profiles and the virulence factors for each country, showing the similarity among the strains, were reported as Supplementary Figure S1.

#### 2.2.1. Argentina

As reported in Table 2, all the 16 Argentinian isolates were positive for a leucocidin (*lukE-lukD*) and for an enterotoxin (*sei*), but negative for the gene encoding exfoliative toxin (*eta*), for *mecA*, *sel* and *sej*. All strains were also negative for two mobile genetic element genes (*chp*, *scn*), while 5 carried *sak*.

Out of 16 isolates, 15 (93.7%) had the genes encoding for *lukE* and *clfA*, 14 (87.5%) for a cell wall-associated protein (*fmtB*), 13 (81.2%) harboured the genes encoding for collagen-binding protein (*cna*), *lukM* and Panton-Valentine leucocidin *lukSF-PV*, whereas 5 (37.5%) were positive for *sak* and/or for *tsst*, respectively.

All the 16 isolates were enterotoxigenic, harbouring at least one of the genes coding for A, C, D, G and H enterotoxins genes. Three isolates from 3 different farms were positive for 5 different enterotoxins (combination of *sea*, *sec*, *seg*, *seh* and *sei* or *sea*, *sed*, *seg*, *seh* and *sei* or *sea*, *sed*, *seg*, *seh* and *sei*) while 8 isolates from 8 different farms were positive for 4 enterotoxins (combination of *sed*, *seg*, *seh* and *sei* or *sea*, *seg*, *seh*). Four isolates, collected in 4 different farms, were positive for 3 enterotoxins genes (combination of *sea*, *seg* and *sei* or *seg*, *seh* and *sei*) and 1 isolates for 2 different enterotoxins genes (*seh*, *sei*).

#### 2.2.2. Brazil

Isolates collected from Brazil were all positive for *fmtB*, *cna*, *clfA* and for the genes encoding leucocidins (*lukE*, *lukE-lukD*, *lukM*, *lukSF-PV*) (Table 3). All the Brazilian isolates were negative for genes carried on mobile genetic elements and usually present in isolates involved in human infections, such as *chp*, *scn*, and *sak*. Moreover, they were negative for *tsst*, *eta*, *mecA*, and *sec*, *sed*, *sel*, *sej*. Out of 15 isolates, 5 (33.3%) were positive for *seh*, 8 (53.3%) for both *sea* and *seh*, while a single isolate (6.6%) harboured other 2 enterotoxin genes (*seg*, *sei*).

#### 2.2.3. Colombia

As shown in Table 4, all the Colombian isolates were positive for *lukE-lukD* and *cna*, but negative for *chp*, *tsst*, *eta*, *mecA* and *sec*, *sel*, *sej*. Out of 15 isolates, 14 (93.4%) were positive for *clfA* and *fmtB* genes, 13 (86.7%) for *lukSF-PV*, 10 (66.7%) for *sak* and *lukM*, and 7 (46.7%) for *scn*. Fourteen (93.3%) isolates were enterotoxigenic harbouring at least one of the genes *sea*, *sed*, *seg*, *sei* or *seh*.

The most frequently detected genes were *seh* (93.3%) and *sea* (86.6%), followed by *sei* (26.6%) and *seg* (20%). One isolate harboured all the 5 enterotoxin genes (*sea*, *sed*, *seg*, *seh* and *sei*); 2 other isolates coming from 2 different farms harboured 4 enterotoxin genes (*sea*, *seg*, *seh* and *sei*) and 1 isolate 3 enterotoxin genes (*sea*, *seh* and *sei*). Finally, 9 isolates, from 6 different farms, had the combination of genes encoding for SEA and SEH.

#### 2.2.4. Germany

All the German isolates were positive for *lukE* and *cna*, but negative for the mobile genetic element genes (*chp*, *scn*, *sak*), for *eta*, *lukSF-PV* and for enterotoxin genes *sed*, *seh*, *sel*, *sej* (Table 5). Out of 17 isolates, one (6%) harboured the *mecA* gene, 4 (23.5%) the *tsst*, 13 (76.5%) the *fmtB*, 15 (88.2%) the *lukM* and 16 (94.1%) both *clfA* and *lukE-lukD* genes.

Fifteen isolates out of 17 (88.2%), collected from 15 different farms, were enterotoxigenic, harbouring at least one of the genes coding for A, C, G and I enterotoxins. The most frequently detected genes were *sea* (88.2%) and *seg* (58.8%), followed by *sei* and *sec* (29.4%). Two isolates harboured all the 4 enterotoxin genes (*sea*, *sec*, *seg*, and *sei*); 3 and 8 other isolates harboured 3 (*sea*, *sec*, and *seg*) or 2 genes (combination of *sea* and *seg*, or *sea* and *sei*), respectively.

#### 2.2.5. Italy

All the Italian isolates were positive for *lukE*, *lukE-lukD*, *cna* and *fmtB*, but negative for *chp*, *eta*, *lukSF-PV* and *seh*, *sel* enterotoxin genes (Table 6). Out of 17 isolates, 14 (82.3%) were positive for *clfA* and 9 (53%) had the gene encoding *lukM*. One isolate (6%) was positive for both *scn* and *sak* genes, and other two different isolates were positive for *tsst* (6%) and *mecA* (6%), respectively.

Fourteen isolates out of 17 (82.3%) were enterotoxigenic, harbouring at least 1 of the genes coding for A, C, D, G, I and J enterotoxins. The most frequently detected genes were *sed* (82.3%) and *seg* (70.5%), followed by *sej* (64.7%), *sea* (58.8%) and *sei* (47%). Six isolates harboured 5 enterotoxin genes (combination of *sea*, *sed*, *seg*, *sei* and *sej,* or *sea*, *sed*, *seg*, *sec* and *sej*); 4 other isolates harboured 4 enterotoxin genes (combination of *sea, sei, sed* and *seg*, or *sei, sed*, *seg* and *sej* or *sea, sed*, *sej* and *seg*). Moreover, 2 isolates harboured 3 different enterotoxins (*sea*, *sed* and *seg*) and 2 isolates, from the same farm, a combination of *sed* and *sej*.

#### 2.2.6. New York State

As reported in Table 7, all the New York State isolates were positive for *lukE-lukD*, but negative for *chp*, *scn*, *sak, tsst*, *eta*, *mecA* and *sec*, *sel*, *seh*, *sej*. Out of 17 isolates, 15 (88.2%) were positive for *cna* and *lukE*, while 13 (76.4%) and 9 (53%) were positive for *lukM* and *clfA* genes, respectively. In addition, 6 isolates (35.2%) and 2 (12%) had the *fmtB* and *lukSF-PV* genes, respectively. Only one isolate was not enterotoxigenic; the remaining 16 isolates (95%) harboured at least one of the genes encoding SEA, SED, SEG, SEI enterotoxins. Five isolates, collected from 5 different farms, had all the enterotoxin genes (*sea*, *sed*, *seg*, *sei*); 6 isolates, from 6 different farms, harboured 3 genes (combination of *sea*, *sed* and *seg* or *sea*, *seg* and *sei* or *sed*, *seg* and *sei*). Five isolates, from 4 different farms, had 2 enterotoxin genes (combination of *sed* and *seg* or *seg* and *sei* or *sed* and *sei*).

#### 2.2.7. South Africa

As reported in Table 8, all the South African isolates were positive for *sak*, *cna*, *lukE-lukD*, *lukE* genes. All the isolates were negative for *chp*, *mecA*, *tsst* and for *sec*, *sed*, *seg*, *sej* and *sel*. In addition, 10 (90.9%) out of 11 isolates were positive for *fmtB*, 7 (63.7%) for *clfA*, 3 (27.3%) for *lukSF-PV*, 2 (18.2%) for *lukM* and 1 (9%) for *eta* genes, respectively. Ten isolates, recovered in 9 different farms, were enterotoxigenic and positive for both *sea* and *seh* genes; out of them, 3 isolates from 2 different farms, harboured also the *sei* gene.

#### 2.2.8. Tunisia

The Tunisian isolates were all positive for *fmtB*, *cna* and *clfA* genes, but negative for *eta*, *mecA*, *lukSF-PV* and *sea*, *sed*, *seg*, *sei*, *sej* (Table 9). Out of 12 isolates, 11 (91.6%) harboured leucocidin genes (*lukM*, *lukE*, *lukE-lukD*). Six isolates (50%) were positive for at least one gene of the immune evasion cluster with the combination of *chp*, *scn* and *sak* for 2 isolates, *scn* and *sak* or *chp* and *scn*, respectively, while the remaining 2 isolates harboured only the *chp* gene. Moreover, 4 isolates from 4 different farms, were enterotoxigenic harbouring *sec* and *sel* (2 isolates) or *seh* genes (2 isolates).

## 3. Discussion

Pathogenic factors of *S. aureus* enable this bacterium to attach, colonize, invade and infect the host tissue. In this study, *S. aureus* isolates, collected from eight different countries, were investigated using RS-PCR genotyping and PCR analysis for the carriage of different virulence factors to examine the epidemiology of this microorganism.

The samples were obtained from collections of the collaborators, allowing a first overview about the presence of the various staphylococcal subtypes among countries. Three new genotypes were observed for South Africa whereas new variants were found in Argentina, Brazil, Colombia, Italy and New York State. As previously described [14], GTB was observed only in Europe (Italy) while CLR and CLC clusters were observed throughout America, Europe and Africa; particularly CLR, which forms a large cluster containing 13 variants, was detected in each country involved, except for Brazil. It is quite well demonstrated [14] that *S. aureus* CLC and CLR clusters are “dairy cattle specific” whose spreading process must have been started a long time ago, with the spreading of breeding cattle from Europe to the other countries. On the contrary, GTB derives from a more recent bovine adaptation due to a new human-to-cow host jump [13]. Certainly, further studies will be necessary to explain the different geographic distribution especially for the minor genotypes.

As previously described [15], *S. aureus* isolates harbouring genes coding for clumping factor (*clfA*), a cell wall-associated protein (*fmtB*), and collagen-binding protein (*cna*) have a greater capability to adhere to extracellular matrix proteins, essential for colonization and the establishment of infections. Our results indicated that, except for the American isolates with a lower presence of *fmtB* and *clfA* genes, in the other seven countries these genes were widely present in the circulating isolates particularly in Brazilian and Tunisian ones. The presence of these genes, necessary for host invasion, could improve the persistence of the microorganism in the host, ensuring the probability of survival in the population.

And more, according to previous studies [11,13,15], except for Brazil, Germany and USA, the remaining countries showed isolates encoding at least 2 virulence factors out of staphylococcal complement inhibitor (*scn*), chemotaxis inhibitory protein of *S. aureus* (*chp*) and staphylokinase (*sak*). These virulence factors show activity prevalently against the human innate immune system but their presence among isolates recovered in herds with high prevalence of *S. aureus* mastitis suggests their involvement also in bovine mammary gland immune response [16], and should be further studied, especially in Colombia and Tunisia where this gene cluster is quite common [17]. In a previous study [4], human strains were grouped in 7 immune evasion cluster (IEC) types, depending on the presence of 2 out of the 3 genes, in association or not with *sea* or *sep*. Unlike Colombian, Italian, South African strains and Tunisian isolates, the Argentinian ones carried only one gene, *sak*, showing a clear distance from human strains. Among the isolates from the other countries, uniquely the Tunisian strains testing positive for IEC, did not harbor *sea*.

Superantigens, especially enterotoxins, have been suggested to play a role in the development of mastitis, for instance by creating an attractive environment for colonization [18] since they are more often identified in *S. aureus* isolated from cows with mastitis than in isolates from healthy cows or from the environment [19]. As a result, enterotoxins support the pathogenesis of *S. aureus* compromising mammary gland immune response and susceptibility to antibiotics resulting in the onset of many diseases [20]. In this study, *sea* and *sei* were the main enterotoxin genes present in all countries except for Tunisia (prevalence between 50% and 90%). While *seh* gene had a frequency higher than 90% in Argentinian, Brazilian, Colombian and South African isolates, *sej* and *sel* genes were carried only by Italian and Tunisian isolates, respectively. Among the 120 isolates analyzed, only 17 (14%) were not enterotoxigenic (1 from Argentina, 1 from Colombia, 2 isolates from Germany, 3 from Italy, 1 from New York State, 1 from South Africa, and 8 from Tunisia). The remaining 103 isolates (86%) harboured a combination of at least 2 up to 5 enterotoxins with the linkages between *sea*, *sed*, *seg* and *seh* confirming their predominance in cows, as previously described [21,22,23,24]. The absence of the enterotoxin genes *seb* and *see* in our isolates was in accordance with previous results [15,22,25,26].

Here, among all the isolates we did not find the presence of *etb* exfoliative gene and only one isolate from South Africa was positive for *eta* gene. These results agree with previous studies conducted in different countries [27,28,29], showing that *S. aureus* isolates from animals with mastitis were rarely positive for exfoliative toxins. On the contrary, in Europe, Kot and coworkers reported a 14.5% of *S. aureus* harbouring the *eta* gene from bovine mastitis [30]. In our study, the presence of *tsst* gene was more relevant, being carried by 37% of Argentinian, 23% of German, 16% of Tunisian and 6% of Italian isolates. All these isolates were also positive at least for a combination of *sec* and *sel*, or *sec*, *seg*, and *sei* or *sec*, *seg* and *sej* or *sec*, *seg* and *sel* genes which are located on the same bovine staphylococcal pathogenicity island (SaPIbov), confirming a positive correlation between *sec, sei* or *sej* and *tsst*, as previously reported [31].

Panton-Valentine leucocidin, encoded by 2 co-transcribed genes located on a prophage, causes leukocyte destruction and tissue necrosis [32]. The presence of PVL-encoding genes in *S. aureus* is reported to be associated with increased disease severity [33]. In the present study, the presence of PVL gene was lower than 20% in South Africa and New York State, higher than 80% in Argentina, Colombia and Brazil, while in Germany, Italy and Tunisia none of the *S. aureus* isolates carried the gene. For European countries, previously published results were in accordance with this study [34,35]. Additionally, genes encoding the bicomponent leucotoxin *lukE-lukD* were observed in all isolates, and, except for South Africa with only 2 isolates, most of the other isolates harboured *lukM*, a gene encoding one operon like the one of PVL. The high rates of *lukE-lukD* and *lukM* found in this study agree with other reports [34,35,36]. Additionally, only 2 isolates, one from Germany and one from Italy were positive for *mec*A, confirming the low diffusion of MRSA among bovine mastitis isolates [37,38]; interestingly, they are both GTS, in accordance with previous results [13].

## 4. Conclusions

Knowledge about the epidemiology of *S. aureus* genotypes in dairy species and herds might help to formulate strategies for reducing the infection spreading and for focused treatments. In our work we found that CLR and CLC clusters and some virulence factors related to host invasion, such as *fmtB*, *cna*, *clfA* or immune defense impairment such as leukocidin genes, were the most frequent ones. These genes combination could be related to the *S. aureus* ability to colonize the host. Further, *fmtB* gene has been shown to be related to the resistance of *S. aureus* to β-lactam antibiotics [10]. Therefore, due to the prevalence of these genes worldwide, it might be useful screening them in *S. aureus* isolates to help predicting clinical outcomes and specially to identify harmful strains. Meanwhile, our work demonstrated also that each country had a specific genotypic pattern and in some countries the isolates harboured some virulence factors, such as PVL-encoding genes, with high prevalence, recommending a close surveillance of *S. aureus* isolates in the animals of these countries to avoid the wide spreading of these genes. Finally, it is notable that most of the isolates worldwide were negative for *mecA*, confirming the evidence of the low diffusion of MRSA among bovine mastitis isolates, as previously described [37,38].

In conclusion, this study confirms the wide variety of *S. aureus* genotypes found in dairy cattle worldwide and that genetic differences are related to geographical origin of the isolates, suggesting that considering the region of interest and the strain virulence might help to formulate strategies directed to reduce the infection spreading and to set up control measures according to pathogen and host features. Therefore, based the characterization of the circulating strain, the farmer would be able to decide to segregate positive cows applying hygienic milking procedures and a suitable milking order, or even to cull the infected animals.

## 5. Materials and Methods

### 5.1. Sample Collection and Bacteriological Analysis

A total of 120 *S. aureus* isolates from eight countries Argentina, Brazil, Colombia, Germany, Italy, New York State, South Africa, Tunisia, (Figure 1), were selected for this study (Table 10). Isolates of *S. aureus* were taken from the authors’ bacterial culture collections (BC) and they included isolates previously collected (Argentina: from April 2015 to June 2017; Brazil: from July 2014 to May 2015; Colombia: from November 2016 to March 2017; Germany: from May 2012 to August 2016; Italy: from September 2012 to December 2016; New York State: from January 2017 to April 2017; South Africa: from August 2016 to February 2017; Tunisia: from September 2015 to December 2016) from clinical mastitis and from high somatic cell count (**H**) samples. The milk collection was made from quarters (**Q**) or composite milk samples (**C**). The isolates were stored at −20 °C until they were transported to the Italian laboratory (University of Milan) where storage was continued at −20 °C until further use. During transport to the laboratory, they were kept frozen using styrofoam boxes and dry ice (for long distances) or wet ice (for short distances).

After samples thawing, 10 μL were streaked on blood agar plate. The plates were then incubated aerobically at 37 °C and examined after 24 h. The colonies were provisionally identified based on morphology and hemolysis patterns and confirmed by coagulase test.

### 5.2. DNA Extraction

DNA was extracted from isolates using the protocol previously described by Cremonesi and co-workers [39]. The amount and quality of DNA were measured using a NanoDrop ND-1000 spectrophotometer (Nano-Drop Technologies, Wilmington, DE, USA), and DNA was stored at −20 °C until use.

### 5.3. Genotyping

All the 120 *nuc* positive isolates (=*S. aureus*) were then genotyped by RS-PCR and a miniaturized electrophoresis system (Agilent Technologies, Santa Clara, CA, USA) as previously described [22,40] where a detailed working protocol is given. The method is based on amplification of the 16S–23S rRNA intergenic spacer region. Each reaction contained (total volume 25 μL) 1× HotStarTaq Master Mix (Qiagen, Hilden Germany), 800 nM of each primer (G1 and L1 primer) [22] and 7 μL of DNA (originally extracted DNA diluted 1:100 in water). The PCR profile was: 95 °C for 15 min, followed by 27 cycles comprising 94 °C for 1 min, followed by a 2 min ramp and annealing at 55 °C for 7 min. After a further 2 min ramp, extension was done at 72 °C for 2 min. PCR was terminated by incubating at 72 °C for 10 min followed by cooling down to 4 °C. One μL of each of the PCR products was then used for the miniaturized electrophoresis (Agilent) performed as described by the manufacturer of the system. New genotypes were named and extended according to Fournier and co-workers [22] leading to the genotypes GTA to GTZ, followed by the genotypes GTAA to GTAZ, GTBA to GTBZ, and GTCA. An electrophoretic pattern differing in one band from the one of a known genotype was considered as a genotypic variant. It was indicated with roman numerals superscripted after the name of the genotype (e.g., GTR^I^, GTR^II^). To identify the genotypes and their variants of the present strains, a freely available, in-house computer program, calculating the corresponding Mahalanobis distance of informative peak sizes and by comparing it to those of the prototype strains using the “Mahalanobis Distances of Staph. aureus Genotypes” software [41]. Finally, genotypes and their variants were combined into genotypic clusters (CL) [14].

### 5.4. Molecular Isolates Characterization

The DNA was amplified to investigate the presence of 26 factors that can contribute in different ways to *S. aureus* pathogenicity and therefore influence the management of the disease. In this study genes encoding enterotoxins (from *sea* to *sel*), leucocidins (*lukE*, *lukSF*-*PV*, *lukE*-*lukD*, *lukM*), the acquisition of methicillin resistance (*mecA*) and genes related to host invasion (*clfA*, *fmtB*, *cna, eta*, *etb*) or to factors that have the potential to interfere with host defense mechanisms (*tsst*, *scn*, *chp*, *sak*) were analyzed using primers and protocols described in literature and listed in Table 11. The amplified PCR fragments were visualized on 2% agarose gel electrophoresis (GellyPhor, Euroclone, Milan, Italy), stained with ethidium bromide (0.05 mg/mL; Sigma Aldrich, Milan, Italy), and visualized by UV transilluminator (BioView Ltd., Nes Ziona, Israel). A 100 bp DNA ladder (Finnzymes, Espoo, Finland) was included in each gel.

Grouping of the RS-PCR profiles and the virulence factors was obtained with the BioNumeric 5.0 software package (Applied Maths, Kortrjik, Belgium) using the UPGMA (unweighted pair group method using arithmetic averages) cluster analysis.

## Figures and Tables

**Table 1:** Distribution of genotypes in the eight countries.

Country	Genotypic Cluster (CL)	Genotype (Isolate No.)	New Genotypes or Variants	Total Strains
	CLC	GTC (6, 15)		
	CLI	GTI^I^ (1, 4, 5, 7)		
		GTI^II^ (10, 11, 14)		
		GTI^V^ (9)		
Argentina		GTI^VI^ (12)	GTI^V^, GTI^VI^	16
	CLP	GTP (8)		
	CLR	GTR^I^ (2, 3, 16)		
		GTR^VI^ (13)		
	CLAQ	GTAQ (31)		
		GTAQ^I^ (30)		
	CLBA	GTBA (17)		
	CLBN	GTBN (29)		
		GTBN^I^ (20)		
		GTBN^II^ (23)		
Brasil	CLBY	GTBY (18, 19, 21, 28)	GTBN^I^, GTBN^II^, GTBY^I^, GTAQ^I^	15
		GTBY^I^ (24, 25)		
	CLC	GTC^III^ (26)		
	CLS	GTS^I^ (22)		
	CLZ	GTZ (27)		
	CLA	GTA^I^ (33)		
	CLAO	GTAO (39, 40, 41)		
		GTAO^I^ (38, 43, 44, 46)		
Colombia		GTAO^II^ (32, 42)	GTAO^I^, GTAO^II^	15
	CLBY	GTBY (45)		
	CLI	GTI^I^ (35, 36, 37)		
	CLR	GTR (34)		
	CLC	GTC^I^ (54, 55, 56, 57, 59)		
	CLR	GTR (47, 48, 49, 51)		
Germany		GTR^I^ (58, 60, 61, 63)		17
		GTR^II^ (50, 62)		
		GTR^VI^ (52)		
	CLS	GTS (53)		
	CLB	GTB (64, 65, 66, 78, 80)		
	CLBG	GTBG (70)		
	CLBQ	GTBQ^I^ (73, 79)		
	CLC	GTC^I^ (69, 75)		
Italy		GTC^II^ (76)	GTR^XIII^	17
	CLR	GTR^I^ (67, 68)		
		GTR^XIII^ (72)		
		GTR^VI^ (71)		
	CLS	GTS (77)		
	CLZ	GTZ (74)		
	CLAI	GTAI (93)		
	CLC	GTC (82, 83, 85, 86, 88, 94, 96)		
		GTC^I^ (81, 87, 91)		
New York State		GTC^III^ (90)	GTC^V^, GTI^V^	17
		GTC^V^ (95)		
	CLI	GTI^I^ (89)		
		GTI^V^ (92, 97)		
	CLR	GTR^I^ (84)		
	CLAR	GTAR (101)		
	CLBH	GTBH (98)		
South Africa	CLBZ	GTBZ (99, 100, 105)	GTAR, GTBZ, GTCA	11
	CLCA	GTCA (103)		
	CLR	GTR (102, 104, 107, 108)		
		GTR^VI^ (106)		
	CLAJ	GTAJ (111)		
	CLBW	GTBW^II^ (110)		
Tunisia	CLCA	GTCA (113, 114)	GTCA	12
	CLCB	GTCB (119)		
	CLR	GTR^I^ (109)		
		GTR^VI^ (112, 115, 116, 117, 118, 120)		

**Table 2:** Molecular characteristics of strains isolated in Argentina.

Isolates	RS-PCR	*clfA*	*fmtB*	*cna*	*lukE*	*lukM*	*lukE-lukD*	*lukSF-PV*	*scn*	*chp*	*sak*	*eta*	*tsst*	Enterotoxins Positive	*mecA*
1	GTI^I^	+	+	+	+	-	+	+	-	-	-	-	+	*sea, seg, sei*	-
2	GTR^I^	+	+	+	+	+	+	+	-	-	+	-	-	*sed, seg, seh, sei*	-
3	GTR^I^	+	-	+	+	+	+	+	-	-	-	-	-	*sed, seg, seh, sei*	-
4	GTI^I^	-	-	+	+	+	+	+	-	-	-	-	-	*sea, seg, sei*	-
5	GTI^I^	+	+	+	+	+	+	+	-	-	-	-	-	*sed, seg, seh, sei*	-
6	GTC	+	+	+	+	+	+	-	-	-	+	-	+	*sea, sec, seg, seh, sei*	-
7	GTI^I^	+	+	+	+	+	+	-	-	-	+	-	+	*sea, seg, seh, sei*	-
8	GTP	+	+	+	-	+	+	+	-	-	+	-	-	*sea, sed, seg, seh, sei*	-
9	GTI^V^ *	+	+	+	+	+	+	+	-	-	-	-	-	*sed, seg, seh, sei*	-
10	GTI^II^	+	+	-	+	+	+	+	-	-	+	-	-	*sea, seg, seh, sei*	-
11	GTI^II^	+	+	-	+	-	+	+	-	-	-	-	-	*sea, seg, seh, sei*	-
12	GTI^VI^ *	+	+	+	+	+	+	+	-	-	-	-	+	*seg, seh, sei*	-
13	GTR^VI^ *	+	+	-	+	+	+	+	-	-	-	-	-	*seg, seh, sei*	-
14	GTI^II^	+	+	+	+	-	+	+	-	-	-	-	-	*sea, sed, seg, seh, sei*	-
15	GTC	+	+	+	+	+	+	+	-	-	-	-	+	*sea, seg, seh, sei*	-
16	GTR^I^	+	+	+	+	+	+	-	-	-	-	-	-	*seh, sei*	-

* new genotypes or new variants.

**Table 3:** Molecular characteristics of strains isolated in Brazil.

Isolates	RS-PCR	*clfA*	*fmtB*	*cna*	*lukE*	*lukM*	*lukE-lukD*	*lukSF-PV*	*scn*	*chp*	*sak*	*eta*	*tsst*	Enterotoxins Positive	*mecA*
17	GTBA	+	+	+	+	+	+	+	-	-	-	-	-	*sea, seh*	-
18	GTBY	+	+	+	+	+	+	+	-	-	-	-	-	*sea, seh*	-
19	GTBY	+	+	+	+	+	+	+	-	-	-	-	-	*seh*	-
20	GTBN^I^ *	+	+	+	+	+	+	+	-	-	-	-	-	*seh*	-
21	GTBY	+	+	+	+	+	+	+	-	-	-	-	-	*seh*	-
22	GTS^I^	+	+	+	+	+	+	+	-	-	-	-	-	*seh*	-
23	GTBN^II^	+	+	+	+	+	+	+	-	-	-	-	-	*seh*	-
24	GTBY^I^	+	+	+	+	+	+	+	-	-	-	-	-	*sea, seh*	-
25	GTBY^I^	+	+	+	+	+	+	+	-	-	-	-	-	*sea, seh*	-
26	GTC^III^	+	+	+	+	+	+	+	-	-	-	-	-	*sea, seh*	-
27	GTZ	+	+	+	+	+	+	+	-	-	-	-	-	*sea, seg, seh, sei*	-
28	GTBY	+	+	+	+	+	+	+	-	-	-	-	-	-	-
29	GTBN	+	+	+	+	+	+	+	-	-	-	-	-	-	-
30	GTAQ^I^	+	+	+	+	+	+	+	-	-	-	-	-	*sea, seh*	-
31	GTAQ	+	+	+	+	+	+	+	-	-	-	-	-	*sea, seh*	-

* new genotypes or new variants.

**Table 4:** Molecular characteristics of strains isolated in Colombia.

Isolates	RS-PCR	*clfA*	*fmtB*	*cna*	*lukE*	*lukM*	*lukE-lukD*	*lukSF-PV*	*scn*	*chp*	*sak*	*eta*	*tsst*	Enterotoxins Positive	*mecA*
32	GTAO^II^ *	-	+	+	+	-	+	+	-	-	-	-	-	-	-
33	GTA^I^	+	+	+	+	+	+	+	+	-	+	-	-	*sea, seh*	-
34	GTR	+	+	+	+	+	+	+	+	-	+	-	-	*seh*	-
35	GTI^I^	+	+	+	+	+	+	+	+	-	+	-	-	*sea, seh*	-
36	GTI^I^	+	+	+	+	-	+	-	+	-	+	-	-	*sea, seh*	-
37	GTI^I^	+	+	+	+	+	+	-	+	-	+	-	-	*sea, seh*	-
38	GTAO^I^ *	+	+	+	+	+	+	+	+	-	+	-	-	*sea, seh*	-
39	GTAO	+	+	+	+	+	+	+	-	-	+	-	-	*sea, seh*	-
40	GTAO	+	+	+	+	+	+	+	-	-	+	-	-	*sea, seh*	-
41	GTAO	+	-	+	+	-	+	+	+	-	+	-	-	*sea, seh*	-
42	GTAO^II^ *	+	+	+	+	+	+	+	-	-	-	-	-	*sea, sed, seg, seh, sei*	-
43	GTAO^I^ *	+	+	+	+	+	+	+	-	-	+	-	-	*sea, seg, seh, sei*	-
44	GTAO^I^ *	+	+	+	+	+	+	+	-	-	-	-	-	*sea, seg, seh, sei*	-
45	GTBY	+	+	+	-	-	+	+	-	-	-	-	-	*sea, seh*	-
46	GTAO^I^ *	+	+	+	+	+	+	+	-	-	-	-	-	*sea, seh, sei*	-

* new genotypes or new variants.

**Table 5:** Molecular characteristics of strains isolated in Germany.

Isolates	RS-PCR	*clfA*	*fmtB*	*cna*	*lukE*	*lukM*	*lukE-lukD*	*lukSF-PV*	*scn*	*sak*	*chp*	*eta*	*tsst*	Enterotoxins Positive	*mecA*
47	GTR	+	+	+	+	+	+	-	-	-	-	-	-	*sea*	-
48	GTR	+	+	+	+	+	+	-	-	-	-	-	-	*sea*	-
49	GTR	+	+	+	+	+	+	-	-	-	-	-	-	*sea, seg*	-
50	GTR^II^	+	+	+	+	+	+	-	-	-	-	-	-	*sea, seg*	-
51	GTR	+	+	+	+	+	+	-	-	-	-	-	-	*sea, seg*	-
52	GTR^VI^	+	+	+	+	-	+	-	-	-	-	-	-	-	-
53	GTS	-	+	+	+	-	-	-	-	-	-	-	-	-	+
54	GTC^I^	+	+	+	+	+	+	-	-	-	-	-	-	*sea, sec, seg, sei*	-
55	GTC^I^	+	-	+	+	+	+	-	-	-	-	-	+	*sea, sec, seg*	-
56	GTC^I^	+	-	+	+	+	+	-	-	-	-	-	+	*sea, sec, seg*	-
57	GTC^I^	+	-	+	+	+	+	-	-	-	-	-	+	*sea, sec, seg, sei*	-
58	GTR^I^	+	+	+	+	+	+	-	-	-	-	-	-	*sea, sei*	-
59	GTC^I^	+	-	+	+	+	+	-	-	-	-	-	+	*sea, sec, seg*	-
60	GTR^I^	+	+	+	+	+	+	-	-	-	-	-	-	*sea, seg*	-
61	GTR^I^	+	+	+	+	+	+	-	-	-	-	-	-	*sea, seg*	-
62	GTR^II^	+	+	+	+	+	+	-	-	-	-	-	-	*sea, sei*	-
63	GTR^I^	+	+	+	+	+	+	-	-	-	-	-	-	*sea, sei*	-

**Table 6:** Molecular characteristics of strains isolated in Italy.

Isolates	RS-PCR	*clfA*	*fmtB*	*cna*	*lukE*	*lukM*	*lukE-lukD*	*lukSF-PV*	*scn*	*chp*	*sak*	*eta*	*tsst*	Enterotoxins Positive	*mecA*
64	GTB	+	+	+	+	-	+	-	-	-	-	-	-	-	-
65	GTB	+	+	+	+	-	+	-	-	-	-	-	-	*sed, sej*	-
66	GTB	-	+	+	+	-	+	-	-	-	-	-	-	*sed, sej*	-
67	GTR^I^	+	+	+	+	-	+	-	-	-	-	-	-	-	-
68	GTR^I^	+	+	+	+	-	+	-	-	-	-	-	-	-	-
69	GTC^I^	+	+	+	+	+	+	-	-	-	-	-	-	*sed, seg, sei, sej*	-
70	GTBG	+	+	+	+	+	+	-	-	-	-	-	-	*sed, seg, sei, sej*	-
71	GTR^VI^	+	+	+	+	+	+	-	-	-	-	-	-	*sea, sed, seg, sei, sej*	-
72	GTR^XIII^ *	+	+	+	+	+	+	-	-	-	-	-	-	*sea, sed, seg, sei*	-
73	GTBQ^I^	+	+	+	+	-	+	-	-	-	-	-	-	*sea, sed, seg, sei, sej*	-
74	GTZ	+	+	+	+	-	+	-	-	-	-	-	-	*sea, sed, seg, sei, sej*	-
75	GTC^I^	+	+	+	+	+	+	-	-	-	-	-	-	*sea, sed, seg*	-
76	GTC^II^	+	+	+	+	+	+	-	-	-	-	-	+	*sea, sec, sed, seg, sej*	-
77	GTS	-	+	+	+	+	+	-	-	-	-	-	-	*sea, sed, seg*	+
78	GTB	+	+	+	+	+	+	-	+	-	+	-	-	*sea, sed, seg, sei, sej*	-
79	GTBQ^I^	+	+	+	+	+	+	-	-	-	-	-	-	*sea, sed, seg, sej*	-
80	GTB	-	+	+	+	-	+	-	-	-	-	-	-	*sea, sed, seg, sei, sej*	-

* new genotypes or new variants.

**Table 7:** Molecular characteristics of strains isolated in New York State.

Isolates	RS-PCR	*clfA*	*fmtB*	*cna*	*lukE*	*lukM*	*lukE-lukD*	*lukSF-PV*	*scn*	*chp*	*sak*	*eta*	*tsst*	Enterotoxins Positive	*mecA*
81	GTC^I^	+	-	+	+	-	+	-	-	-	-	-	-	*sea, sed, seg, sei*	-
82	GTC	-	-	+	+	+	+	-	-	-	-	-	-	*sea, sed, seg, sei*	-
83	GTC	+	+	+	+	+	+	-	-	-	-	-	-	*sed, seg*	-
84	GTR^I^	+	+	+	+	-	+	-	-	-	-	-	-	*sed, seg*	-
85	GTC	+	-	+	+	+	+	-	-	-	-	-	-	*sed, seg, sei*	-
86	GTC	+	-	+	+	+	+	-	-	-	-	-	-	*seg, sei*	-
87	GTC^I^	+	-	+	+	+	+	-	-	-	-	-	-	*sea, sed, seg*	-
88	GTC	+	-	+	+	+	+	-	-	-	-	-	-	*sea, sed, seg, sei*	-
89	GTI^I^	+	+	+	+	+	+	-	-	-	-	-	-	*sed, seg, sei*	-
90	GTC^III^	-	-	-	-	+	+	-	-	-	-	-	-	*sea, sed, seg*	-
91	GTC^I^	-	-	+	+	+	+	-	-	-	-	-	-	*sea, sed, seg, sei*	-
92	GTI^V^ *	-	+	+	+	+	+	-	-	-	-	-	-	*sed, sei*	-
93	GTA^I^	+	-	+	-	-	+	-	-	-	-	-	-	*sea, sed, seg, sei*	-
94	GTC	-	-	+	+	+	+	-	-	-	-	-	-	*sea, seg, sei*	-
95	GTC^V^ *	-	-	-	+	+	+	+	-	-	-	-	-	*sea, seg, sei*	-
96	GTC	-	+	+	+	+	+	-	-	-	-	-	-	*seg, sei*	-
97	GTI^V^ *	-	+	+	+	-	+	+	-	-	-	-	-	-	-

* new genotypes or new variants.

**Table 8:** Molecular characteristics of strains isolated in South Africa.

Isolates	RS-PCR	*clfA*	*fmtB*	*cna*	*lukE*	*lukM*	*lukE-lukD*	*lukSF-PV*	*scn*	*chp*	*sak*	*eta*	*tsst*	Enterotoxins Positive	*mecA*
98	GTBH	+	+	+	+	-	+	+	-	-	+	-	-	*sea, seh*	-
99	GTBZ *	-	+	+	+	-	+	-	-	-	+	-	-	*sea, seh, sei*	-
100	GTBZ *	-	+	+	+	-	+	-	-	-	+	-	-	*sea, seh, sei*	-
101	GTAR *	-	+	+	+	-	+	-	-	-	+	-	-	*sea, seh, sei*	-
102	GTR	+	+	+	+	+	+	-	-	-	+	-	-	*sea, seh*	-
103	GTCA *	+	+	+	+	-	+	-	+	-	+	+	-	*sea, seh*	-
104	GTR	+	+	+	+	-	+	-	-	-	+	-	-	*sea, seh*	-
105	GTBZ *	-	-	+	+	-	+	-	-	-	+	-	-	*sea, seh*	-
106	GTR^VI^	+	+	+	+	+	+	-	-	-	+	-	-	*sea, seh*	-
107	GTR	+	+	+	+	-	+	+	-	-	+	-	-	*sea, seh*	-
108	GTR	+	+	+	+	-	+	+	-	-	+	-	-	-	-

* new genotypes or new variants.

**Table 9:** Molecular characteristics of strains isolated in Tunisia.

Isolates	RS-PCR	*clfA*	*fmtB*	*cna*	*lukE*	*lukM*	*lukE-lukD*	*lukSF-PV*	*scn*	*chp*	*sak*	*eta*	*tsst*	Enterotoxins Positive	*mecA*
109	GTR^I^	+	+	+	-	-	+	-	-	-	-	-	-	-	-
110	GTBW^II^	+	+	+	+	+	+	-	-	-	-	-	+	*sec, sel*	-
111	GTAJ	+	+	+	+	+	+	-	-	-	-	-	+	*sec, sel*	-
112	GTR^VI^	+	+	+	+	+	+	-	+	+	+	-	-	-	-
113	GTCA	+	+	+	+	+	+	-	-	+	-	-	-	*seh*	-
114	GTCA	+	+	+	+	+	+	-	+	-	+	-	-	*seh*	-
115	GTR^VI^	+	+	+	+	+	+	-	-	-	-	-	-	-	-
116	GTR^VI^	+	+	+	+	+	+	-	-	+	-	-	-	-	-
117	GTR^VI^	+	+	+	+	+	+	-	-	-	-	-	-	-	-
118	GTR^VI^	+	+	+	+	+	+	-	-	-	-	-	-	-	-
119	GTCB	+	+	+	+	+	+	-	+	+	-	-	-	-	-
120	GTR^VI^	+	+	+	+	+	-	-	+	+	+	-	-	-	-

**Table 10:** World survey on *S. aureus* cow isolates: participating countries, total isolates analyzed per country, number of isolated from clinical mastitis or high somatic cell count (H) samples, and type of sample collection (C = composite milk sample; Q = quarter milk sample).

Country	Total Isolates Analyzed per Country			
	Clinical Mastitis	High SCC (H)	Number of Farms	Type of Sample
**Argentina**	16		10	C
**Brazil**	15		12	Q
**Colombia**		15	11	Q
**Germany**	17		17	Q
**Italy**	17		15	Q
**New York State (USA)**	17		13	Q
**South Africa**	11		9	Q
**Tunisia**		12	10	C
**Total**	93	27	97	

**Table 11:** Primer used in this study for *S. aureus* isolates characterization.

Target Gene	Primer Sequence (5′-3′)	Amplification Size	Reference
Invasion			
*clf*A	GGCTTCAGTGCTTGTAGG	1000 bp	[42]
	TTTTCAGGGTCAATATAAGC		
*coa*	CCGCTTCAACTTCAGCCTAC	204 bp	[43]
	TTAGGTGCTACAGGGGCAAT		
*nuc*	AGTTCAGCAAATGCATCACA	400 bp	[43]
	TAGCCAAGCCTTGACGAACT		
*luk*E	AATGTTAGCTGCAACTTTGTCA	831 bp	[22]
	CTTTCTGCGTAAATACCAGTTCTA		
*luk*M	TGGATGTTACCTATGCAACCTAC	780 bp	[44]
	GTTCGTTTCCATATAATGAATCACTAC		
*luk*E-*luk*D	TGAAAAAGGTTCAAAGTTGATACGAG	269 bp	[44]
	TGTATTCGATAGCAAAAGCAGTGCA		
*lukSF-PV*	ATCATTAGGTAAAATGTCTGGACATGATCA	433 bp	[45]
	GCATCAAGTGTATTGGATAGCAAAAGC		
*scn*	ATACTTGCGGGAACTTTAGCAA	320 bp	[10]
	TTTTAGTGCTTCGTCAATTTCG		
*chp*	TTTTTAACGGCAGGAATCAGTA	404 bp	[10]
	TGCATATTCATTAGTTTTTCCAGG		
*fmtB*	AATGAAGATGCGAATCATGTTG	725 bp	[10]
	CATCCATTTTTGTTTGCGTAGA		
*sak*	TGAGGTAAGTGCATCAAGTTCA	403 bp	[10]
	CCTTTGTAATTAAGTTGAATCCAGG		
*cna*	AAAGCGTTGCCTAGTGGAGA	192 bp	[46]
	AGTGCCTTCCCAAACCTTTT		
Interfere with host defense mechanism			
*tsst*	ATGGCAGCATCAGCTTGATA	300 bp	[42]
	TTTCCAATAACCACCCGTTT		
*eta*	CTAGTGCATTTGTTATTCAA	120 bp	[42]
	TGCATTGACACCATAGTACT		
*etb*	ACGGCTATATACATTCAATT	200 bp	[42]
	TCCATCGATAATATACCTAA		
*sea*	TAAGGAGGTGGTGCCTATGG	180 bp	[43]
	CATCGAAACCAGCCAAAGTT		
*seb*	TCGCATCAAACTGACAAACG	478 bp	[44]
	GCAGGTACTCTATAAGTGCC		
*sec*	ACCAGACCCTATGCCAGATG	371 bp	[43]
	TCCCATTATCAAAGTGGTTTCC		
*sed*	TCAATTCAAAAGAAATGGCTCA	339 bp	[43]
	TTTTTCCGCGCTGTATTTTT		
*see*	TACCAATTAACTTGTGGATAGAC	170 bp	[47]
	CTCTTTGCACCTTACCGC		
*seg*	CCACCTGTTGAAGGAAGAGG	432 bp	[43]
	TGCAGAACCATCAAACTCGT		
*seh*	TCACATCATATGCGAAAGCAG	463 bp	[43]
	TCGGACAATATTTTTCTGATCTTT		
*sei*	CTCAAGGTGATATTGGTGTAGG	529 bp	[43]
	CAGGCAGTCCATCTCCTGTA		
*sej*	GGTTTTCAATGTTCTGGTGGT	306 bp	[43]
	AACCAACGGTTCTTTTGAGG		
*sel*	CACCAGAATCACACCGCTTA	240 bp	[43]
	CTGTTTGATGCTTGCCATTG		
Antibiotic resistance			
*mecA*	GTAGAAATGACTGAACGTCCGATAA	310 bp	[45]
	CCAATTCCACATTGTTTCGGTCTAA		

## Supplementary Materials

The following are available online at http://www.mdpi.com/2072-6651/10/6/247/s1, Figure S1: Unweighted pair-group method with arithmetic averages (UPGMA)-based dendrogram derived from the combined RS-PCR profiles and the virulence factors of the *S. aureus* strains considered in this study.

## References

[1] Deb R., Kumar A., Chakraborty S., Verma A.K., Tiwari R., Dhama K., Singh U., Kumar S. (2013). Trends in diagnosis and control of bovine mastitis: A review. Pak. J. Biol. Sci..

[2] Gomes F., Henriques M. (2016). Control of Bovine Mastitis: Old and Recent Therapeutic Approaches. Curr. Microbiol..

[3] Foster T.J. (2005). Immune evasion by staphylococci. Nat. Rev. Microbiol..

[4] Van Wamel W.J., Rooijakkers S.H., Ruyken M., van Kessel K.P., van Strijp J.A. (2006). The innate immune modulators staphylococcal complement inhibitor and chemotaxis inhibitory protein of *Staphylococcus aureus* are located on beta-hemolysin-converting bacteriophages. J. Bacteriol..

[5] Pantosti A. (2012). Methicillin-Resistant *Staphylococcus aureus* associated with animals and its relevance to human health. Front. Microbiol..

[6] Sawant A.A., Sordillo L.M., Jayarao B.M. (2005). A survey on antibiotic usage in dairy herds in Pennsylvania. J. Dairy Sci..

[7] Fitzgerald J.R., Meaney W.J., Hartigan P.J., Smyth C.J., Kapur V. (1997). Fine-structure molecular epidemiological analysis of *Staphylococcus aureus* recovered from cows. Epidemiol. Infect..

[8] Sommerhäuser J., Kloppert B., Wolter W., Zschöck M., Sobiraj A., Failing K. (2003). The epidemiology of *Staphylococcus aureus* infections from subclinical mastitis in dairy cows during a control programme. Vet. Microbiol..

[9] Herron-Olson L., Fitzgerald J.R., Musser J.M., Kapur V. (2007). Molecular correlates of host specialization in *Staphylococcus aureus*. PLoS ONE.

[10] Sung J.M., Lloyd D.H., Lindsay J.A. (2008). *Staphylococcus aureus* host specificity: Comparative genomics of human versus animal isolates by multi-strain microarray. Microbiology.

[11] Ikawaty R., Brouwer E.C., Jansen M.D., van Duijkeren E., Mevius D., Verhoef J., Fluit A.C. (2009). Characterization of Dutch *Staphylococcus aureus* from bovine mastitis using a Multiple Locus Variable Number Tandem Repeat Analysis. Vet. Microbiol..

[12] Zadoks R.N., Middleton J.R., McDougall S., Katholm J., Schukken Y.H. (2011). Molecular epidemiology of mastitis pathogens of dairy cattle and comparative relevance to humans. J. Mammary Gland Biol. Neoplasia.

[13] Cremonesi P., Pozzi F., Raschetti M., Bignoli G., Capra E., Graber H.U., Vezzoli F., Piccinini R., Bertasi B., Biffani S. (2015). Genomic characteristics of *Staphylococcus aureus* strains associated with high within-herd prevalence of intramammary infections in dairy cows. J. Dairy Sci..

[14] Cosandey A., Boss R., Luini M., Artursson K., Bardiau M., Breitenwieser F., Hehenberger E., Lam T., Mansfeld M., Michel A., Mösslacher G. (2016). *Staphylococcus aureus* genotype B and other genotypes isolated from cow milk in European countries. J. Dairy Sci..

[15] Cremonesi P., Zottola T., Locatelli C., Pollera C., Castiglioni B., Scaccabarozzi L., Moroni P. (2013). Identification of virulence factors in 16S-23S rRNA intergenic spacer genotyped *Staphylococcus aureus* isolated from water buffaloes and small ruminants. J. Dairy Sci..

[16] Magro G., Biffani S., Minozzi G., Ehricht R., Monecke S., Luini V., Piccinini R. (2017). Virulence genes of *S. aureus* from dairy cow mastitis and contagiousness risk. Toxins.

[17] Ben Said M., Abbassi M.S., Bianchini V., Sghaier S., Cremonesi P., Romanò A., Gualdi V., Hassen A., Luini M.V. (2016). Genetic characterization and antimicrobial resistance of *Staphylococcus aureus* isolated from bovine milk in Tunisia. Lett. Appl. Microbiol..

[18] Piccinini R., Borromeo V., Zecconi A. (2010). Relationship between *Staphylococcus aureus* gene pattern and dairy herd mastitis. Vet. Microbiol..

[19] Piechota M., Kot B., Zdunek E., Mitrus J., Wicha J., Wolska M.K., Sachanowicz K. (2014). Distribution of classical enterotoxin genes in staphylococci from milk of cows with- and without mastitis and the cowshed environment. Pol. J. Vet. Sci..

[20] El-Sayed A., Alber J., Lammler C., Jager S., Woter W., Vázquez H. (2006). Comparative study on genotypic properties of *Staphylococcus aureus* isolated from clinical and subclinical mastitis in Mexico. Vet. Mex..

[21] Haveri M., Roslöf A., Rantala L., Pyörälä S. (2007). Virulence genes of bovine *Staphylococcus aureus* from persistent and nonpersistent intramammary infections with different clinical characteristics. J. Appl. Microbiol..

[22] Fournier C., Kuhnert P., Frey J., Miserez R., Kirchhofer M., Kaufmann T., Steiner A., Graber H.U. (2008). Bovine *Staphylococcus aureus*: Association of virulence genes, genotypes and clinical outcome. Res. Vet. Sci..

[23] Artursson K., Söderlund R., Liu L., Monecke S., Schelin J. (2016). Genotyping of *Staphylococcus aureus* in bovine mastitis and correlation to phenotypic characteristics. Vet. Microbiol..

[24] Sharma V., Sharma S., Dahiya D.K., Khan A., Mathur M., Sharma A. (2017). Coagulase gene polymorphism, enterotoxigenecity, biofilm production, and antibiotic resistance in *Staphylococcus aureus* isolated from bovine raw milk in North West India. Ann. Clin. Microbiol. Antimicrob..

[25] Bystroń J., Bania J., Lis E., Molenda J., Bednarski M. (2009). Characterisation of *Staphylococcus aureus* strains isolated from cows’ milk. Bull. Vet. Inst. Pulawy.

[26] Ote I., Taminiau B., Duprez J.N., Dizier I., Mainil J.G. (2011). Genotypic characterization by polymerase chain reaction of *Staphylococcus aureus* isolates associated with bovine mastitis. Vet. Microbiol..

[27] Darwish S.F., Asfour H.A. (2013). Investigation of biofilm forming ability in Staphylococci causing bovine mastitis using phenotypic and genotypic assays. Sci. World J..

[28] Silveira-Filho V.M., Luz I.S., Campos A.P., Silva W.M., Barros M.P., Medeiros E.S., Freitas M.F., Mota R.A., Sena M.J., Leal-Balbino T.C. (2014). Antibiotic resistance and molecular analysis of *Staphylococcus aureus* isolated from cow’s milk and dairy products in northeast Brazil. J. Food Prot..

[29] Akindolire M.A., Babalola O.O., Ateba C.N. (2015). Detection of Antibiotic Resistant *Staphylococcus aureus* from Milk: A Public Health Implication. Int. J. Environ. Res. Public Health.

[30] Kot B., Szweda P., Frankowska-Maciejewska A., Piechota M., Wolska K. (2016). Virulence gene profiles in *Staphylococcus aureus* isolated from cows with subclinical mastitis in eastern Poland. J. Dairy Res..

[31] Zschöck M., Kloppert B., Wolter W., Hamann H.P., Lammler C.H. (2005). Pattern of enterotoxin genes seg, seh, sei and sej positive *Staphylococcus aureus* isolated from bovine mastitis. Vet. Microbiol..

[32] Bhatta D.R., Cavaco L.M., Nath C., Kumar K., Gaur A., Gokhale S., Bhatta D.R. (2016). Association of Panton Valentine leukocidin (PVL) genes with methicillin-resistant *Staphylococcus aureus* (MRSA) in Western Nepal: A matter of concern for community infections (a hospital based prospective study). BMC Infect. Dis..

[33] Shariati L., Validi M., Hasheminia A.M., Ghasemikhah R., Kianpour F., Karimi A., Nafisi M.R., Tabatabaiefar M.A. (2016). *Staphylococcus aureus* isolates carrying Panton-Valentine leucocidin genes: Their frequency, antimicrobial patterns, and association with infectious disease in Shahrekord city, Southwest Iran. Jundishapur J. Microbiol..

[34] Fueyo J.M., Mendoza M.C., Rodicio M.R., Muñiz J., Alvarez M.A., Martín M.C. (2005). Cytotoxin and pyrogenic toxin superantigen gene profiles of *Staphylococcus aureus* associated with subclinical mastitis in dairy cows and relationships with macrorestriction genomic profiles. J. Clin. Microbiol..

[35] Parisi A., Caruso M., Normanno G., Latorre L., Sottili R., Miccolupo A., Fraccalvieri R., Santagada G. (2016). Prevalence, antimicrobial susceptibility and molecular typing of methicillin-resistant *Staphylococcus aureus* (MRSA) in bulk tank milk from southern Italy. Food Microbiol..

[36] Schlotter K., Ehricht R., Hotzel H., Monecke S., Pfeffer M., Donat K. (2012). Leukocidin genes lukF-P83 and lukM are associated with *Staphylococcus aureus* clonal complexes 151, 479 and 133 isolated from bovine udder infections in Thuringia, Germany. Vet. Res..

[37] Luini M., Cremonesi P., Magro G., Bianchini V., Minozzi G., Castiglioni B., Piccinini R. (2015). Methicillin-resistant *Staphylococcus aureus* (MRSA) is associated with low within-herd prevalence of intra-mammary infections in dairy cows: Genotyping of isolates. Vet. Microbiol..

[38] Hendriksen R.S., Mevius D.J., Schroeter A., Teale C., Meunier D., Butaye P., Franco A., Utinane A., Amado A., Moreno M. (2008). Prevalence of antimicrobial resistance among bacterial pathogens isolated from cattle in different European countries: 2002–2004. Acta Vet. Scand..

[39] Cremonesi P., Castiglioni B., Malferrari G., Biunno I., Vimercati C., Moroni P., Morandi S., Luzzana M. (2006). Technical Note: Improved method for rapid DNA extraction of mastitis pathogens directly from milk. J. Dairy Sci..

[40] Graber H.U. (2016). Genotyping of *Staphylococcus aureus* by Ribosomal Spacer PCR (RS-PCR). J. Vis. Exp..

[41] Syring C., Boss R., Reist M., Bodmer M., Hummerjohann J., Gehrig P., Graber H.U. (2012). Bovine mastitis: The diagnostic properties of a PCR-based assay to monitor the *Staphylococcus aureus* genotype B status of a herd, using bulk tank milk. J. Dairy Sci..

[42] Akineden O., Annemuller C., Hassan A.A., Lammler C., Wolter W., Zschock M. (2001). Toxin genes and other characteristics of *Staphylococcus aureus* isolates from milk of cows with mastitis. Clin. Diagn. Lab. Immunol..

[43] Cremonesi P., Luzzana M., Brasca M., Morandi S., Lodi R., Vimercati C., Agnellini D., Caramenti G., Moroni P., Castiglioni B. (2005). Development of a multiplex PCR assay for the identification of *Staphylococcus aureus* enterotoxigenic strains isolated from milk and dairy products. Mol. Cell. Probes.

[44] Jarraud S., Mougel C., Thioulouse J., Lina G., Meugnier H., Forey F., Nesme X., Etienne J., Vandenesch F. (2001). Relationships between *Staphylococcus aureus* genetic background, virulence factors, *agr* groups (alleles), and human disease. Infect. Immun..

[45] McClure J.A., Conly J.M., Lau V., Elsayed S., Louie T., Hutchins W., Zhang K. (2006). Novel multiplex PCR assay for detection of the staphylococcal virulence marker Panton-Valentine leukocidin genes and simultaneous discrimination of methicillin-susceptible from -resistant staphylococci. J. Clin. Microbiol..

[46] Zecconi A., Cesaris L., Liandris E., Dapra V., Piccinini R. (2006). Role of several *Staphylococcus aureus* virulence factors on the inflammatory response in bovine mammary gland. Microb. Pathog..

[47] Monday S.R., Bohach G.A. (1999). Use of multiplex PCR to detect classical and newly described pyrogenic toxin genes in staphylococcal isolates. J. Clin. Microbiol..

