# Supplementary material for: Staphylococcus aureus Isolates from Bovine Mastitis in Eight Countries: Genotypes, Detection of Genes Encoding Different Toxins and Other Virulence Genes
Source: Toxins (Basel). 2018 Jun 17;10(6):247. doi: 10.3390/toxins10060247 (PMC6024761; doi:10.3390/toxins10060247)
Supplement: Supplementary file 1 [file toxins-10-00247-s001.pdf]

Supplementary Materials: *Staphylococcus aureus* Isolates from Bovine Mastitis in Eight Countries: Genotypes, Detection of Genes Encoding Different Toxins and Other Virulence Genes

Valentina Monistero, Hans Ulrich Graber, Claudia Pollera, Paola Cremonesi, Bianca Castiglioni, Enriqueta Bottini, Alejandro Ceballos-Marquez, Laura Lasso-Rojas, Volker Kroemker, Nicole Wente, Inge-Marie Petzer, Carlos Santisteban, Jeff Runyan, Marcos Veiga dos Santos, Bruna Gomes Alves, Renata Piccinini, Valerio Bronzo, Mohamed Salah Abbassi, Meriam Ben Said and Paolo Moroni

Unweighted pair-group method with arithmetic averages (UPGMA)-based dendrogram derived from the combined RS-PCR profiles and the virulence factors of the *S. aureus* strains considered in this study.

Argentina

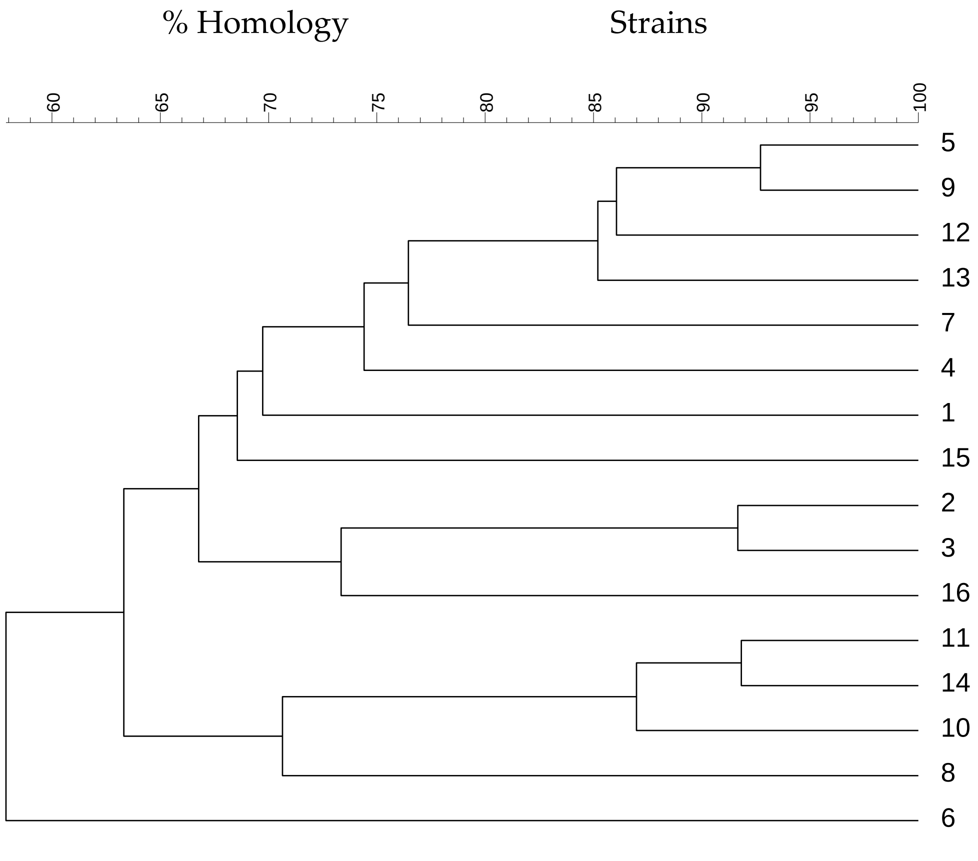

Brazil

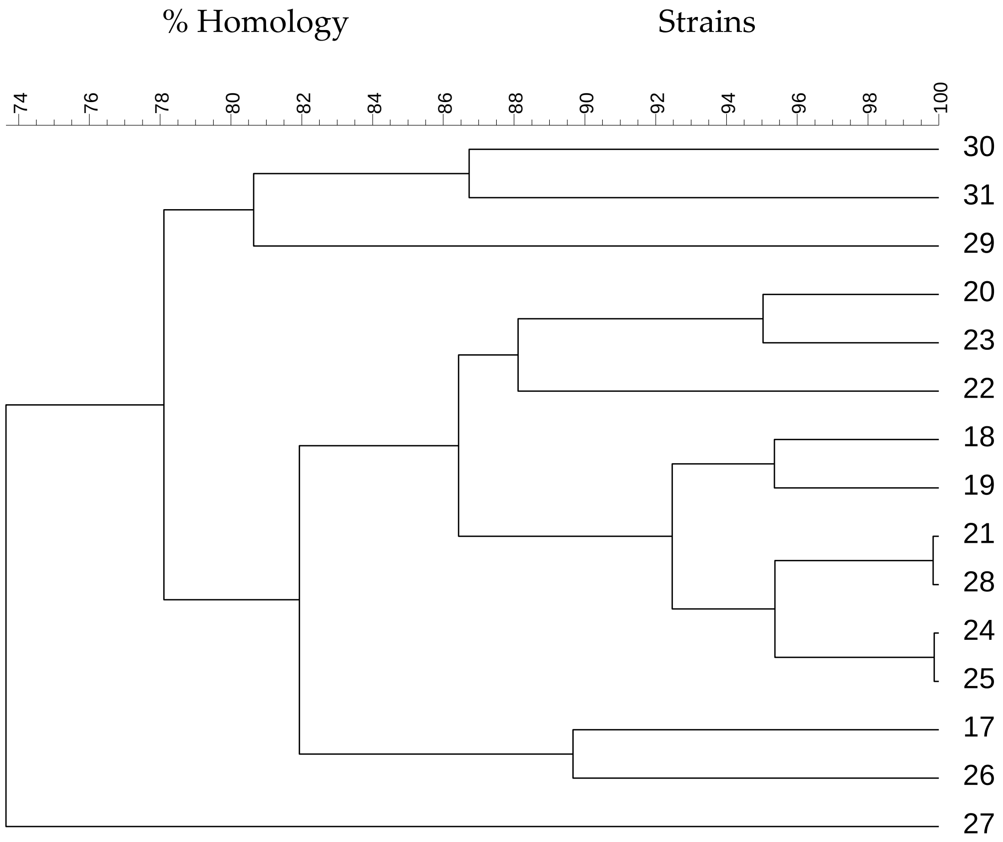
% Homology
Strains
74
76
78
80
82
84
86
88
90
92
94
96
98
100
30
31
29
20
23
22
18
19
21
28
24
25
17
26
27

Colombia

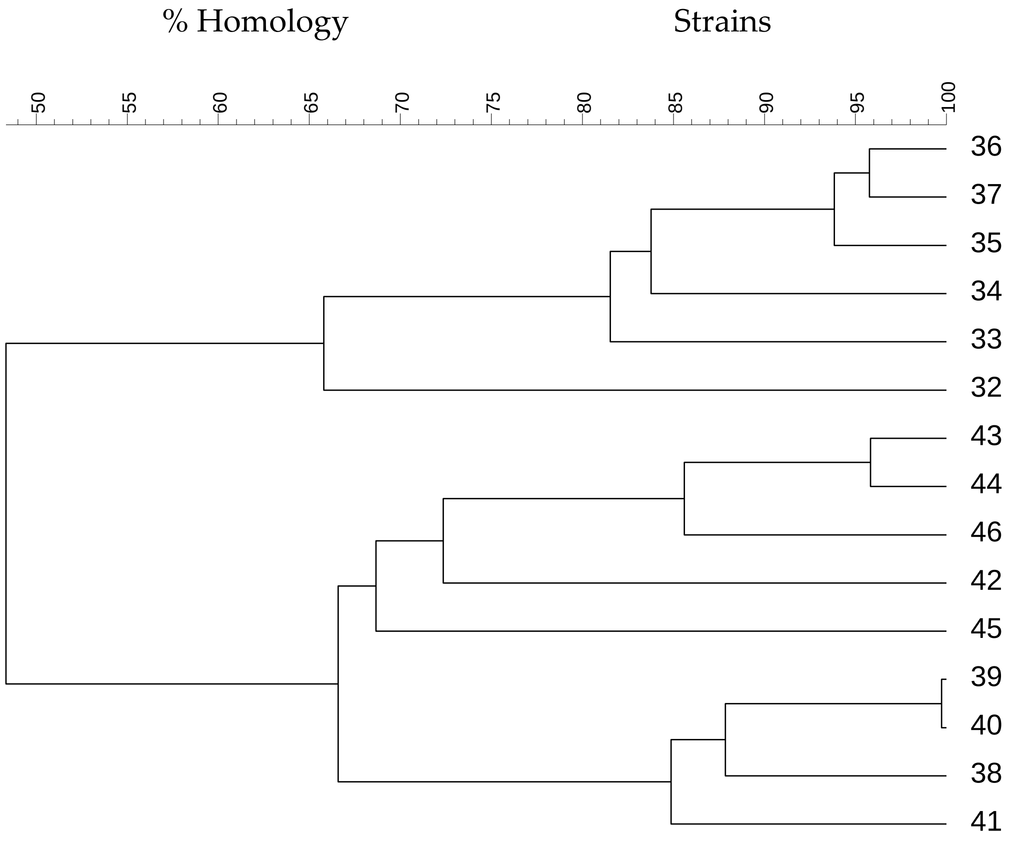
% Homology
Strains
50
55
60
65
70
75
80
85
90
95
100
36
37
35
34
33
32
43
44
46
42
45
39
40
38
41

Germany

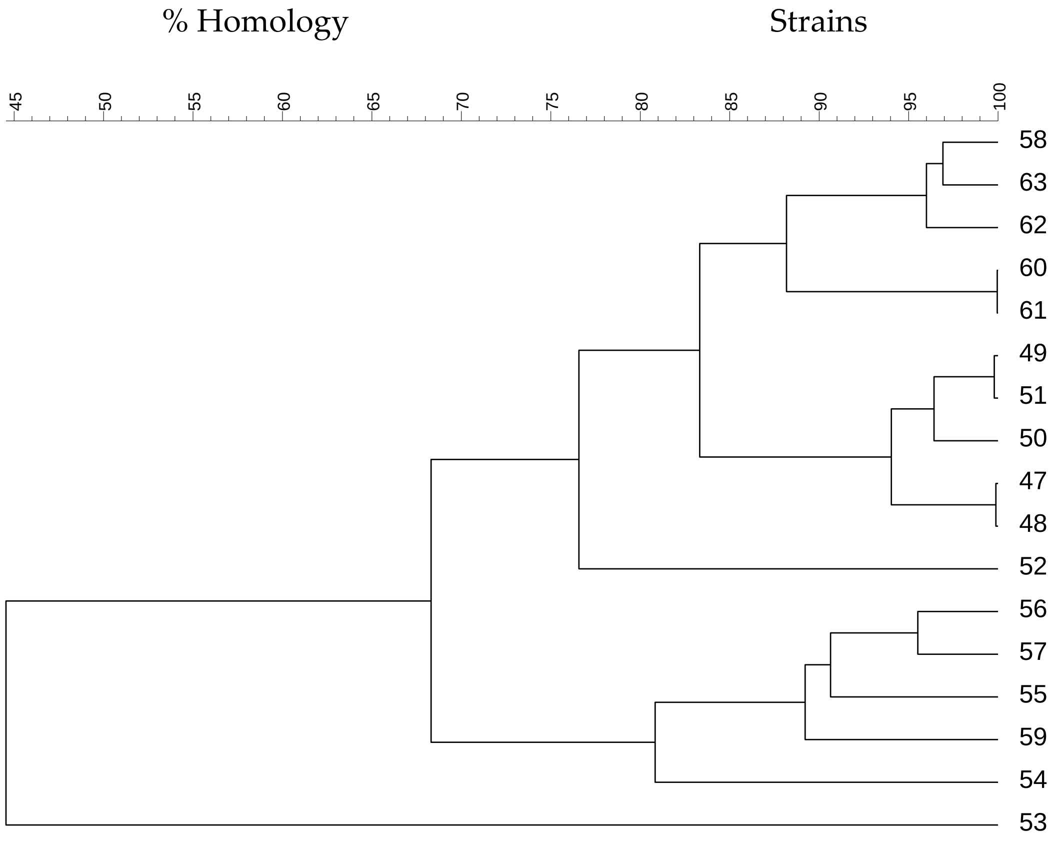
% Homology
Strains
45
50
55
60
65
70
75
80
85
90
95
100
58
63
62
60
61
49
51
50
47
48
52
56
57
55
59
54
53

Italy

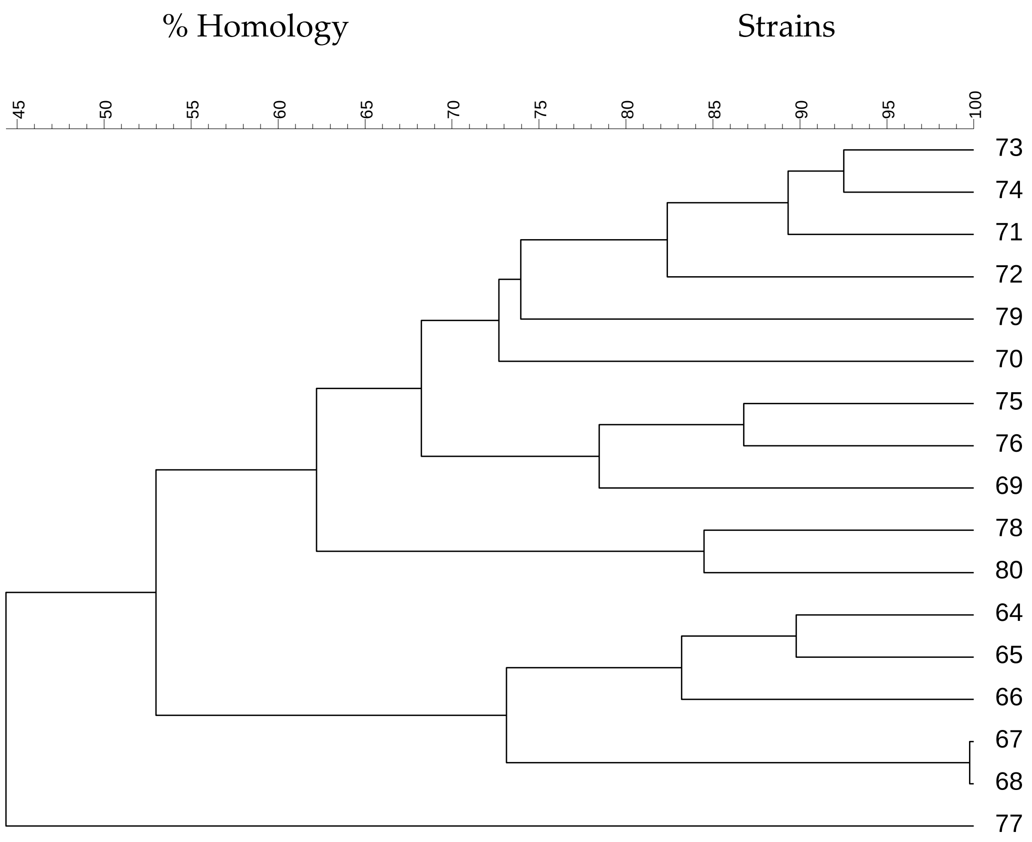
% Homology
Strains
45
50
55
60
65
70
75
80
85
90
95
100
73
74
71
72
79
70
75
76
69
78
80
64
65
66
67
68
77

New York State

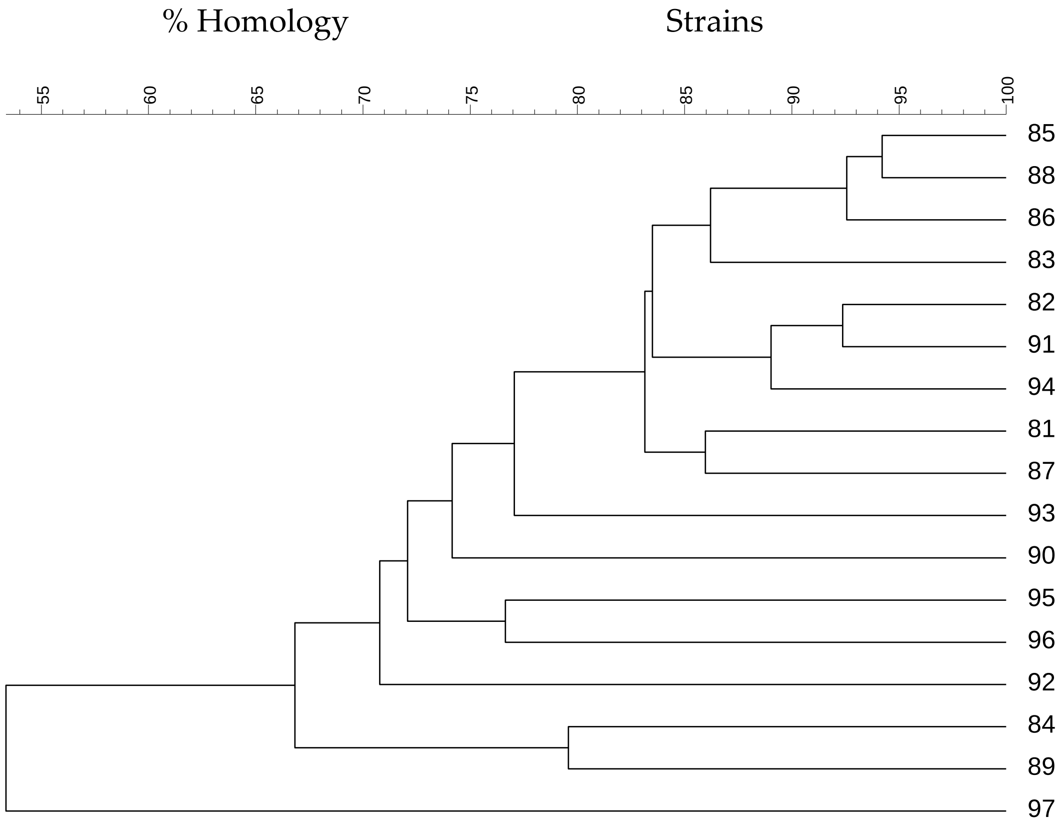

South Africa

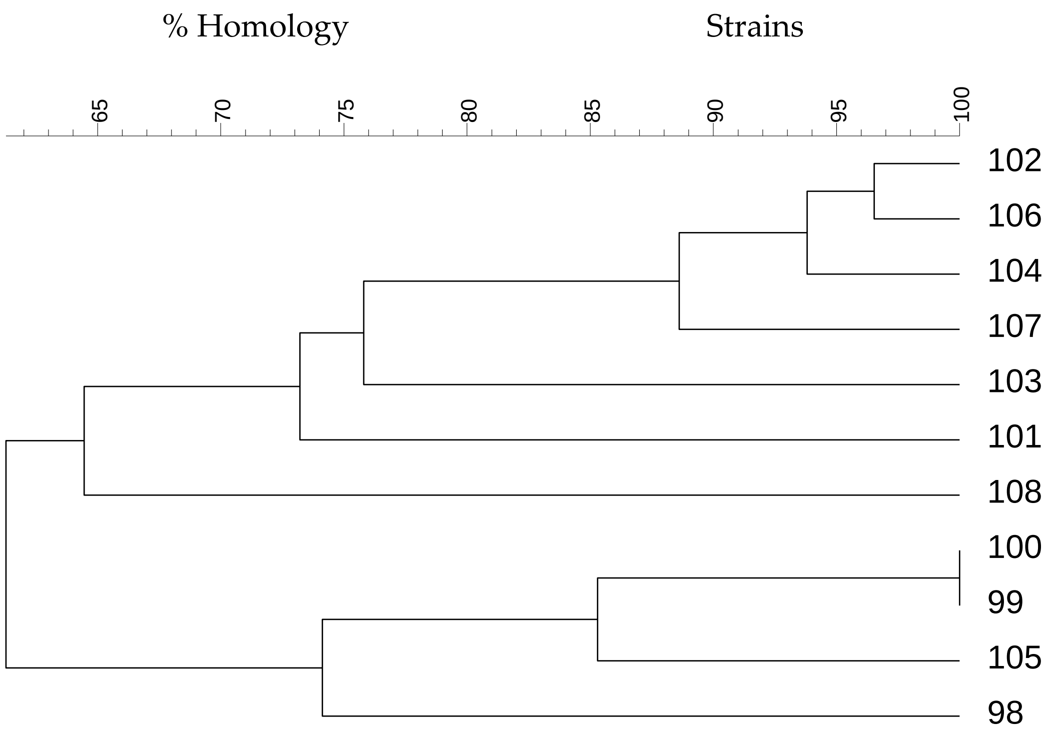

Tunisia

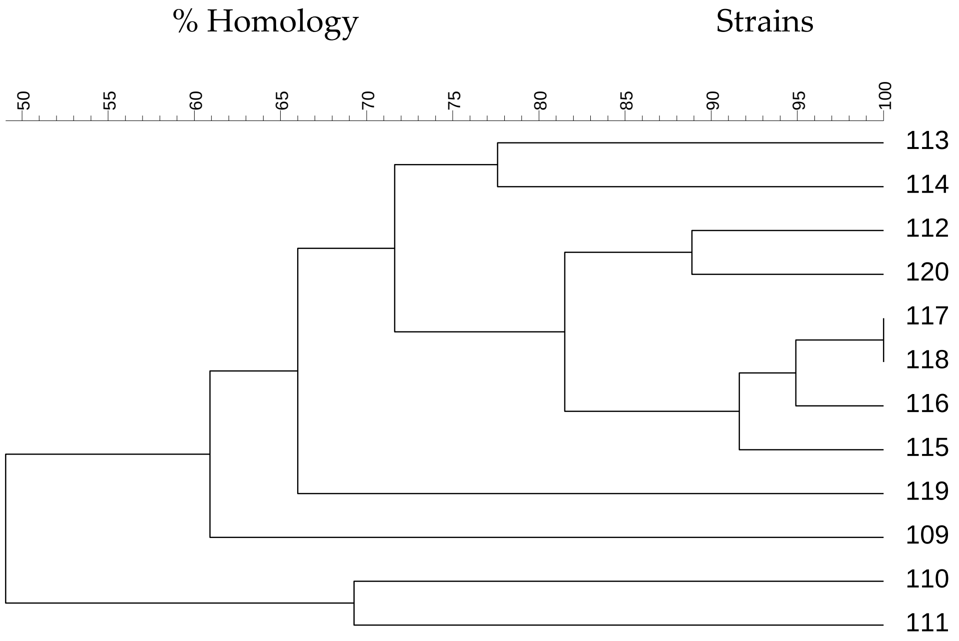
% Homology
Strains
50
55
60
65
70
75
80
85
90
95
100
113
114
112
120
117
118
116
115
119
109
110
111
